# Mortality of Surgical Operations in the Upper Lake States, Compared with That of Other Regions

**Published:** 1876-07

**Authors:** Edmund Andrews

**Affiliations:** Professor of Principles and Practice of Surgery in Chicago Medical College


### Chicago Medical Journal

AND

### EXAMINER.

Vol. XXXIII. — JULY, 1876. — No. 7.

### Original Communications.

### THE MORTALITY OF SURGICAL OPERATIONS IN THE UPPER LAKE STATES, COMPARED WITH THAT OF OTHER REGIONS.

By EDMUND ANDREWS, A.M., M.D.,

Professor of Principles and Practice of Surgery in Chicago Medical College,

Assisted by Thos. B. Lacey, M.D.,

Assistant Surgeon in the National Soldiers' Home.

Operative Surgery in the Lake States of America has results widely different from those of the Atlantic region, and of Europe. Many operations are much less fatal here than there, so that to the most important of all questions about a proposed operation, viz., What is its danger? the Western practitioner can find no book to furnish him a correct answer. Full proof of this will be given as we proceed.

The object of the present essay is to assist the Western surgeon in ascertaining with regard to the principal operations:

1. What is their risk in the Lake States?
2. What has it been in other regions?
3. What are the opinions and precepts of the principal surgeons of the world regarding each?
4. What conclusions are we to draw for our own guidance?

Before entering upon details, we may illustrate the wide difference between the results of our surgery and that of other regions by studying the following condensed table:

TABLE I.

¶ Statist. des Hopitaux de Paris, 1861—63.

** Aerztlicher Bericht, k. k. allgemeinen Krankenhauses, Wien, 1861—71.

The first thing which strikes the Western surgeon in this table, is the prodigious excess of mortality reported almost everywhere. With us the average mortality of all the four major amputations combined, is only 20 per cent., while in the hospitals of the Atlantic States it is 30 per cent. ; in the great Imperial General Hospital (k. k. allg. Krankenhaus) of Vienna, 36 per cent., in the British hospitals, 41 per cent., and in the famous hospitals of Paris, it attains the astounding figure of 60 per cent.

Yet these rates of mortality are not inevitable results in each region, for if we examine more closely, we find that the British country hospitals have a mortality but little greater than those of Chicago.

I pass over, for the present, the strange figures of Sir J. Y. Simpson about British private practice, which show an apparent mortality for the four major amputations, of only 10 per cent. The error of these figures has been exposed by Callender and others, and the curious way in which it occurred will be hereafter explained.

Again, if we take the important operation of herniotomy, we find a perfectly similar result, as we may see from the following figures :

TABLE II.

*Showing the Mortality of Herniotomy, in the Lake States, compared with that of other Regions.*

| | CASES. | DEATHS. | PER CT. MORT. |
|---|---|---|---|
| Lake States | 34 | 8 | 24 |
| Vienna General Hospital | 259 | 114 | 44 |
| London Hospitals | 326 | 136 | 42 |
| British larger Provincial Hospitals | 177 | 72 | 41 |
| " smaller " " | 118 | 53 | 45 |
| Paris Hospitals | 361 | 244 | 68 |
| Combined Hospital and Private Practice in Boston, Cheever | 27 | 14 | 52 |

It thus appears that the very hospitals and great masters to which we have resorted most for instruction in surgery have the least success in curing their patients. It follows also, that as operative risks with us thus differ greatly from the rates generally given at the East, we shall be compelled to revise all the estimates, and deduce new rules adapted to the facts of our own region. It is in the hope to contribute something to this great end, that the following facts and figures have been laboriously collected.

It will be remarked by referring to Table I, that there is but little difference in the Lake States between the results of private and of hospital practice, and, I may also add, between country and city practice. Here are the figures, fractions being omitted:

| | | |
|---|---|---|
| Mort. of the 4 maj. amp's in | Lake States Hospitals | 19 per ct. |
| " " " | private practice in the Lake States | 19 " |
| " " " | country " " | 20 " |

In contrast to this approximate uniformity, Sir J. Y. Simpson represents the mortality of hospital major amputations in Great Britain to be four times as great as in private country practice, viz.:

| | |
|---|---|
| British civil hospital mortality | 41 per cent. |
| " private country practice mortality | 11 " |

Two things here surprise the Western American surgeon:

1. That the difference between the hospital and private practice is so enormous—

2. That country amputations in Great Britain should be twice as successful as among the vigorous, well-fed population around our great lakes.

This last point requires consideration, and certainly looks like an error, for our country people are robust, well fed, and well housed, probably more so than the British peasantry. It cannot be the difference in skill, for though the average American grade of professional education is lamentably low, yet the country surgeons, from whom I collected these cases were picked men, all known to me to be men of education, and generally of superior capacity. Taking them together, I pronounce them, without hesitation, to be fully equal to country surgeons in Great Britain.

After careful reflection, I think that Sir J. Y. Simpson's country statistics are grossly delusive, and that he in all honesty has fallen into a fatal error in the manner of collecting his cases. I wish to dwell upon this a little, because the same identical blunder is repeated every year by committees of medical societies, desirous of collecting surgical statistics for their reports.

Sir J. Y. Simpson printed a quantity of blank reports of amputations to be filled out with the operations and their results, and mailed these to an immense number of country surgeons, most of whom must have been personally unknown to him. Now, in the replies thus obtained, there will be three sources of error, all tending to understate the deaths and exaggerate the proportion of recoveries.

1. Those surgeons who are honest, but have chanced to have a "run of bad luck," that is, an accidental series of incurable cases, will not be likely to answer the circular. They are chagrined at the results of their efforts, and indisposed to court publicity for them. To a large extent these men will neglect to reply, and their fatal cases will be lost to the collector.

2. For analogous reasons the honest surgeon, who has had accidentally a "run" of favorable cases, feels exhilarated, and quite desirous to have them brought to notice. Such men will all respond, and thus give a preponderance of successful cases.

3. The dishonest men, (and there are perhaps some liars in Great Britain, as well as elsewhere) look upon the circular as a favorable opportunity to bring themselves into the notice, at least of Sir James, and perhaps of the rest of the world, if he should chance to publish names. They will therefore fill up the paper with false cases, or true cases with false results, or deceive more gracefully by omitting their fatal cases.

It is inevitable that statistics gathered by promiscuous circulars must be grossly delusive, and they always falsify on the successful side.

Impressed with the necessity of avoiding this error, I only applied to men of known and high-toned honesty, and almost always made my application in such a way as to secure a positive response. In this way the number of cases collected was much smaller than that obtained by Simpson, but they are truthful, and, I believe, represent correctly the results of operative surgery in this region.

If this correction could be made in Sir James' tables, I think the results of British private practice would not differ greatly from the American.

There is another mode of collecting statistics equally absurd which it may be worth while to mention here. This consists in searching the files of medical journals and picking up and tabulating the operations there recorded. The notorious fact that men for the most part go into print with only successful cases, is sufficient to show the utter worthlessness of such figures.

I have not entered upon the old dispute whether statistics are of any use. Fifteen years ago the principal English surgeons were accustomed either to scout them openly, or, when they employed them, entered a protest that they attached little weight to them. At the present time, the whole surgical world is agreed, that properly collected, they are important aids in arriving at truth.

The fact is, statistics are simply recorded experience, and cannot be ignored, any more than experience in any other form. They have the same liability to error as other methods of investigation, viz., that they may be unskillfully or dishonestly managed, and fail to reach the truth ; but the same must be said of all other modes of recording experience. No sane man will pin his faith to statistics alone, but all surgeons at the present day recognize them as important aids, in our methods of research.

The cases in this essay are derived—

1. From the surgical records of Mercy Hospital, which have been carefully preserved under my own supervision since June, 1859.

2. From records of my private practice.

3. From such partial records of operations in the Marine, the County, St. Luke's and the two Women's Hospitals, as survived the great fire.

4. Notes of the operations of surgeons in Chicago and in the surrounding country, who are personally known to me, and whose statements I believe can be relied on for candor and truth.

As I desire that these statistics shall be worthy of the highest confidence, I have carefully rejected from the Lake States lists all matter furnished by persons not known to be trustworthy, as well as all tables of cases, published in journals or in reports of societies, whose mode of compilation was not fully known to me. The tables of Western practice here given, embody with absolute impartiality the failures as well as the successes of the operators, so that they may be trusted as fair samples of the operative work of this region.

In collating, for comparison with ours, the printed statistics of other regions, I have, so far as possible, rejected all figures collected by the faulty methods above referred to, but it is impossible to determine the faithfulness and honesty of distant authors with the same precision that we can that of our own acquaintances.

All military cases are omitted from the tables of Lake States surgery, but are often quoted for comparison in stating results elsewhere. There are several excellent surgeons in Chicago, such as Professors Freer, Gunn, Isham, Sherman, Powell and Bogue, who were unable to furnish me more than a very few cases, notwithstanding their extensive experience, partly because they lost all their papers in the great fire, which swept the city, and partly because the hospitals, in which they served, lost their books in the same tremendous conflagration. Otherwise the tables might have been more extensive, though the ratios of mortality would not have been materially changed.

The hospital cases are almost entirely derived from the records of Mercy Hospital, and it is worthy of notice that, contrary to the experience elsewhere, their results have equaled those of private practice. I attribute this good fortune mainly to the special care which I have given to ventilation and other antiseptic measures.

AMPUTATIONS.

First among these we will consider the disarticulations of the shoulder joint, of which very few have occurred in this city. In my own practice, I have uniformly preferred excision of the joint wherever the choice was possible, because it is not only less dangerous but leaves a very useful limb.

### TABLE III.

*Amputations at the Shoulder Joint.*

| Operator. | Age. | Reason for Operation. | Complications. | Operation. | General Condition at time of Operation. | Time from beginning of case to Operation. | Result. | Time from Operation to Death or Recovery. | Hospital or Priv. Practice. |
|---|---|---|---|---|---|---|---|---|---|
| Dr. E. Andrews | | Comp. fract. humerus from R. R. cars | Tissues of chest torn | Flap. | | Primary. | Recovered. | 42 days. | Hospital. |
| " A. Fisher | 30 yrs. | Gunshot fracture at joint | | | Medium. | 7 days. | " | | " |
| " Hooper | 32 " | R. R. fracture humerus | None | | Good. | 3 " | Died.† | 36 hrs. | " |
| " J. H. Hollister | 24 " | Caries of humerus after ampt. at mid. 3d | None | | Bad. | | Recovered. | | Priv. Prac. |
| Cook Co. Hospital* | | Disease of parts | | | | | Died. | | Hospital. |
| Dr. A. J. Baxter | 36 " | Necrosis after fract. humerus | None | | Bad. | 6 weeks. | " | | Priv. Prac. |
| " A. J. Baxter | 25 " | Compound fract. humerus | None | | Good. | Primary. | Recovered. | | " |
| " A. J. Baxter | 35 " | Compound fract. shoulder | None | | Medium. | " | " | | " |
| " A. J. Baxter | | Necrosis of humerus after ampt. of arm | None | | " | 6 months | " | | " |
| " A. J. Baxter | | Compound fract. of humerus | | | " | Primary. | " | | " |
| " E. W. Lee | 50 " | Compound fract. of humerus | | | Bad. | | " | 4 weeks | " |
| " E. W. Lee | 45 " | Compound fracture of humerus | | Flap. | " | Primary. | " | 5 " | " |
| " S. Marks | 34 " | Traumat. aneurism of subclavian artery | | | Medium. | 8 weeks. | " | 4 " | Hospital. |

* Surgeon's name not recorded. † Cause of death, pyæmia.

Total, 13 cases.

Recapitulation—Recovered, 10, of which 5 were primary, 2 secondary, 1 pathological, and 2 not stated.
Died, 3, of which 2 were secondary, and 1 pathological. General mortality, 23 per cent.
Hospital practice, 5 cases, of which 2 died. Private practice, 8 cases, of which 1 died.

### DISARTICULATIONS OF THE SHOULDER ABROAD.

I find the following records of this operation in various countries :

### TRAUMATIC PRIMARY CASES.

| AUTHORITIES. | CASES. | DEATHS. |
|---|---|---|
| New York Hospital, Boston Med. Jour., 1872 | 7 | 4 |
| Boston City Hosp. Rept., Dr. Cheever | 9 | 7 |
| Mass. Gen. Hosp., 1871 | 15 | 8 |
| Penn. Hosp., Dr. Norris | 11 | 2 |
| U. S. Marine Hosp. Repts., Dr. Woodworth | 1 | 1 |
| St. Thomas Hosp. Rept., London | 2 | 1 |
| St. Bartholomew's Hosp., London, Mr. Callender | 2 | 0 |
| St. George's Hosp., London | 3 | 3 |
| K. k. allg. Krankenhaus, Wien. | 8 | 4 |
| Dr. Herrgalt, Strasburg | 1 | 1 |
| Leeds Gen. Infirmary, Mr. Nunnely | 9 | 4 |
| Glasgow Infirmary, Glasgow Med. Jour., 1854 | 19 | 8 |
| Siege of Antwerp, Schmidt's Jahrbücher, vol. 156, p. 249 | 5 | 0 |
| Paris, 1830—32—48, " " " " | 2 | 1 |
| Crimean War, " " " " | 172 | 105 |
| Italian " " " " " | 12 | 5 |
| British Mil. Hosp., Brussels, 1815, Guthrie's Com. | 6 | 1 |
| Schleswig Holstein War, Schmidt's Jahrbücher, vol. 156 | 6 | 3 |
| War of 1866, " " " | 2 | 0 |
| Battles of Vittoria, Pyrenees and St. Sebastian, Guthrie, quoted in Dict. des Sci. Med., Art. Amputations | 19 | 1 |
| Totals | 311 | 159 |

Mortality, 51 per cent.

### TRAUMATIC SECONDARY CASES.

| | CASES. | DEATHS. |
|---|---|---|
| New York Hosp., Boston Med. Jour., 1872 | 4 | 2 |
| Boston City Hosp. Rept., Dr. Cheever | 2 | 1 |
| Billroth's Letters | 1 | 0 |
| Dr. Herrgalt, Strasburg | 1 | 1 |
| Battles of Vittoria, Pyrenees, and St. Sebastian, Guthrie, quoted in Dict. des Sci. Med., Art. Amputations | 19 | 15 |
| Glasgow Infirmary, Glasgow Med. Jour., 1854 | 7 | 4 |
| Siege of Antwerp, Schmidt's Jahrbücher, vol. 156 | 3 | 2 |
| Paris, 1830—32—48, " " " | 2 | 1 |
| Crimean War, " " " | 56 | 35 |
| Italian War, " " " | 34 | 16 |
| Schleswig Holstein War, " " " | 4 | 3 |
| War of 1866, " " " | 9 | 3 |
| Dutch and German War, " " " | 4 | 1 |
| British Mil. Hosp. in Brussels, 1815, Guthrie's Com. | 12 | 6 |
| Totals | 158 | 90 |

Mortality, 57 per cent.

CASES WITH TIME OR CAUSE NOT STATED.

| AUTHORITIES. | CASES. | DEATHS. |
|---|---|---|
| Rostock Hosp. Rept., 1868 | 2 | 0 |
| St. Bartholomew's Hosp., 1863—71 | 1 | 0 |
| Circular No. 6, Surgeon Gen. U. S. A. | 237 | 93 |
| " 3, " " | 4 | 1 |
| Trans. Ill. State Med. Soc., 1863 | 6 | 2 |
| Statist. des Hôpitaux de Paris, 1861—63 | 7 | 6 |
| Deutsches Zeitschrift für Chir., Bd. 2, S. 380 | 4 | 2 |
| Arch. Klin. Chir., Bd. 13 | 2 | 0 |
| Siege of Antwerp, Schmidt's Jahrbücher, vol. 156 | 8 | 2 |
| Paris, 1830—32—48, " " " | 14 | 5 |
| Crimean War, " " " | 261 | 152 |
| Italian War, " " " | 166 | 92 |
| Schleswig Holstein War, " " " | 20 | 9 |
| War of 1866, " " " | 14 | 5 |
| Dutch and German War, " " " | 29 | 16 |
| Lehrbuch Resectionen von O. Heyfelder, p. 220 | 222 | 101 |
| Totals | 997 | 486 |

Mortality, 49 per cent.

PATHOLOGICAL CASES.

| AUTHORITIES. | CASES. | DEATHS. |
|---|---|---|
| New York Hospital, Boston Medical Journal, 1872 | 2 | 1 |
| Boston City Hospital, reported by Dr. Cheever | 1 | 0 |
| Massachusetts Gen. Hosp., Boston Medical Jour., 1872 | 11 | 3 |
| Pennsylvania Hospital, " " " " | 1 | 0 |
| Guy's Hospital Reports | 1 | 1 |
| St. Thomas' Hospital Reports | 1 | 0 |
| St. Bartholomew's Hospital Reports, 1863—71 | 5 | 0 |
| Statist. des Hôp. de Paris, 1861—63 | 6 | 3 |
| Archiv. Klin. Chir., Bd. 8 and 10 | 4 | 1 |
| Leeds General Infirmary, Mr. Nunnely | 2 | 1 |
| Totals | 34 | 10 |

Mortality, 29 per cent.

SUMMARY OF SHOULDER AMPUTATIONS ABROAD.

| | CASES. | DEATHS. | PER CENT. MORTALITY. |
|---|---|---|---|
| Traumatic, primary | 311 | 159 | 51 |
| " secondary | 158 | 90 | 57 |
| Time or cause not stated | 997 | 86 | 49 |
| Pathological | 34 | 10 | 29 |
| Totals | 1,500 | 745 | 50 |

Mortality in the Lake States, 23 per cent.

From these figures it appears that the mortality of this operation abroad averages 50 per cent., which is more than twice that observed in the Lake States.

The primary amputations are a little less fatal than the secondary, but pathological cases are everywhere the safest, being only 29 per cent. abroad, and in the Lake States still less.

All the shoulder amputations are more fatal than resection, hence amputation should only be performed when resection is inadmissible. We shall discuss the relative dangers more fully when we come to treat of resections. At present it suffices to say that the operation is not justifiable for such cases as mere caries, compound fracture of the joint, etc., etc., for which it is occasionally performed.

OPINIONS OF AUTHORS.

Demme's statistics show that for gunshot fractures of the shoulder, excision gives the best, and conservative treatment the worst, results.

Dr. A. Kadis (Petersburg Med. Paper, 1869) says resection gives the best results, and disarticulation should be used only in the worst, and conservative treatment in the lightest cases; excision to be employed when the bone is comminuted and likely to become carious.

Joseph Lister says (Holmes' Surg., vol. V, p. 637): "Amputation at the shoulder-joint * * * yields very satisfactory results."

T. Holmes says (Holmes' Surg., vol. V, p. 664) amputation is only to be performed for injuries of the shoulder too extensive for excision, but is to be preferred for rapidly growing tumor of the head of the bone, especially if cancerous, but never for anchylosis.

Gant's Surgery, p. 289, says that in bullet wounds of the shoulder, amputation is not equal to excision; p. 454, speaking of compound fractures of the head of the humerus, that "amputation must be resorted to in any additional injuries to the vessels and nerves;" p. 673, says amputation is less favorable than excision by about 6 per cent.

Guthrie advocated amputation at the shoulder for gunshot fractures of the upper part of the shaft of the humerus.

Erichsen (Surgery, vol. I, p. 203) advises to resect such cases, unless important vessels and nerves are destroyed also.

Ashurst (p. 117) says the results of this amputation are "tolerably favorable."

### CONCLUSIONS.

1. Amputation at the shoulder in the Lake States has a mortality only half that stated in the books.
2. It may be practiced when the parts below are so destroyed by violence or invaded by cancer as to admit of no more distal operation.
3. It should not be practiced for caries, for gunshot fractures not involving the great vessels and nerves, nor for any other condition which admits of resection.

### TABLE IV.

*Amputations of the Arm.*

| Operator. | Age. Yrs. | Reason for Operation. | Complications. | Operation. | General Condition at time of Operation. | Time from beginning of case to Operation. | Result. | Time from Operation to Death or Recovery. | Hospital or Priv. Practice. |
|---|---|---|---|---|---|---|---|---|---|
| Dr. E. Andrews |  | Arm crushed by cars |  | Middle 3d. |  | Primary. | Recovered | 28 days. | Hospital. |
| " E. Andrews |  | Arm crushed by machinery |  | Both arms at 2 & 6 in. below shoulder |  | " | " |  | Priv. Prac. |
| " E. Andrews |  | Gunshot fract. of arm |  | Middle 3d. |  | Not sta'd | " |  | " |
| " E. Andrews | 7 | Arm crushed by cars | Leg crushed | Flap, upper 3d. |  | Primary. | Died. | 48 hrs. | Hospital. |
| " E. Andrews |  | Necrosis of stump of humerus | None | Re-ampt. mid. 3d. |  | 15 mos. | Recovered | 50 days. | " |
| " E. Andrews | 25 | Ununited fract. from gunshot wound | None | Upper 3d. | Good. | 2 years. | " |  | Priv. Prac. |
| " E. Andrews | 36 | Arm lacerated by machinery | None |  | " | Primary. | " | 4 mos. | Hospital. |
| " E Andrews* | 24 | Cancer of stump | None |  | Med. |  | " |  | " |
| " E. Andrews† | 25 | Arm torn off by machinery | None | Circ., upper 3d. | " | Primary. | " |  | " |
| " E. Andrews | 70 | Cancer | None | Upper 3d. | Bad. |  | " |  | " |
| " La Count | 45 | Forearm torn off by machinery | None | Circ., middle 3d. | Good. | Primary. | " |  | Priv. Prac. |
| " La Count | 45 | Mortification of forearm after fract. | Abscess in arm | Flap, upper 3d. | " | Second'y | " |  | " |
| " La Count | 25 | Forearm torn off at elbow by machinery | None | " " | " | Primary. | " |  | " |
| Cook Co. Hospital |  | Injury |  |  |  | " | " |  | Hospital. |
| Cook Co. Hospital |  | Injury |  |  |  | Second'y | " |  | " |
| Dr. H. Wardner | 12 | Compound fract. forearm | Tight band. and mort. | Upper 3d. | Bad. | 3 days. | " ‡ | 5 weeks | Priv. Prac. |
| " H. Wardner | 12 | Arm crushed by R. R. | Chest injured severely | " | " | Primary. | Died. § | 3 days. | " |
| " H. Wardner | 13 | Compound fract. elbow | Erysipelas & gangrene | Lower 3d. | " | 2 weeks. | Recovered | 4 weeks | " |
| " E. D. Kittoe | 14 | Necrosis bones of arm and forearm | None | Upper 3d. | " | 11 mos. | " |  | " |
| " E. D. Kittoe | 24 | Member mortified by tight binding | " | Middle 3d. | " | Second'y | " |  | " |
| " E. D. Kittoe | 23 | Gunshot wound of elbow and arm | Great shock | Upper 3d. | Good. | Primary. | " |  | " |
| " E. W. Lee | 10 | Compound fract. humerus |  | Flap, upper 3d. | " | " | " | 3 weeks | " |
| " E. W. Lee | 20 | Compound fract. humerus |  | " " | Med. | " | " | 4 " | " |
| " S. Marks | 26 | Traumatic gangrene |  | Upper 3d. | " | 10 days. | " | 4 " | Hospital. |
| " S. Marks | 21 | Compound fract. humerus | Tetanus | " | Bad. | 20 hours. | Died. |  |  |
| " S. Marks | 30 | Compound fract. humerus |  | Lower 3d. | Good. | Primary. | Recovered | 6 weeks | Priv. Prac. |
| " S. Marks | 5 | Compound fract. humerus |  | Upper 3d. | " | " | " | 18 days. | " |

* No. in Andrews' Surgical Record, 8,399.

† No. in Andrews' Surgical Record, 8,421.

‡ Had tetanus 20 days.

§ Cause of death, gangrene.

RECAPITULATION.

| | CASES. | DEATHS. | PER CENT. MORTALITY. |
|---|---|---|---|
| Total Number | 27 | 3 | 11 |
| Traumatic, primary | 15 | 3 | 20 |
| " secondary | 8 | 0 | 0 |
| Time of operation not stated | 1 | 0 | 0 |
| Pathological | 3 | 0 | 0 |
| Hospital Cases | 10 | 1 | 10 |
| Private Practice | 16 | 1 | 6 |

AMPUTATION OF THE ARM ABROAD.

The following figures give a fair view of the world's experience in this operation :

TRAUMATIC PRIMARY.

| AUTHORITIES. | CASES. | DEATHS. |
|---|---|---|
| British Mil. Hosp. in Brussels, 1815, Guthrie's Com'ntaries. | 21 | 4 |
| American War of Secession, Confed. Army, Warren, of N.C. | 92 | 16 |
| New York Hosp., Bost. Med. Jour., May 1, 1872 | 14 | 0 |
| Pennsylvania Hosp., " " " " | 58 | 5 |
| Boston City " " " " " | 14 | 4 |
| Mass. Gen. " " " " " | 36 | 7 |
| Guy's Hosp. Reports, London | 15 | 6 |
| St. Thomas' Hosp., " | 2 | 1 |
| St. Bartholomew's Hosp., " 1853—71 | 45 | 4 |
| St. George's " " | 3 | 2 |
| Mr. Richardson, at Birmingham, England, 1853—64 | 32 | 12 |
| Various German Surgeons, Franco-German War | 22 | 10 |
| Mr. Nunnely, Leeds Gen. Infirmary, England | 62 | 22 |
| Crimean War, Schmidt's Jahrbücher, Bd. 156, S. 249 | 849 | 489 |
| Dr. Löffler, Danish War with Prussia | 19 | 9 |
| Dr. Beck, at Tauberbischofsheim | 7 | 2 |
| Siege of Antwerp, Schmidt's Jahrbücher, Bd. 156, S. 249 | 9 | 1 |
| Franco-German War, " " " " | 40 | 19 |
| Schleswig-Holstein War, " " " " | 19 | 9 |
| War of 1866, " " " " | 7 | 2 |
| Totals | 1,366 | 624 |

Mortality, 46 per cent.

TRAUMATIC SECONDARY.

| AUTHORITIES. | CASES. | DEATHS. |
|---|---|---|
| New York Hospital, Bost. Med. Jour., May, 1872 | 4 | 1 |
| Pennsylvania " " " " " | 9 | 3 |
| Boston City " " " " " | 8 | 5 |
| Mass. Gen. " " " " " | 8 | 3 |
| Guy's " London | 12 | 7 |
| St. Thomas' " " | 2 | 0 |
| St. Bartholomew's Hosp., London, 1853—71 | 29 | 9 |
| St. George's " " | 3 | 3 |
| Mr. Richardson, Birmingham, 1853—64 | 15 | 2 |
| Various German Surgeons, Franco-German War | 16 | 9 |
| British Army in the Crimea | 16 | 6 |
| Schleswig-Holstein War, Dr. Löffler | 12 | 8 |
| Dr. Beck, at Tauberbischofsheim | 14 | 3 |
| Maas and Billroth, each a case | 2 | 2 |
| Seige of Antwerp, Schmidt's Jahrbücher, Bd. 156, S. 249 | 2 | 1 |
| Crimean War, " " " " | 146 | 86 |
| Franco-German War, " " " " | 31 | 21 |
| Schleswig-Holstein War" " " " | 12 | 8 |
| War of 1866, " " " " | 15 | 4 |
| American War of Secession, Confed. Army, Warren | 100 | 38 |
| British Mil. Hosp., Brussels, 1815, Guthrie's Commentaries | 51 | 13 |
| Totals | 507 | 232 |

Mortality, 46 per cent.

TRAUMATIC, TIME NOT STATED.

| AUTHORITIES. | CASES. | DEATHS. |
|---|---|---|
| Circular No. 6, Surg. Gen. U. S. A. | 1,949 | 414 |
| " " 3, " " " | 24 | 5 |
| Schleswig-Holstein War, Esmarch | 54 | 19 |
| Lucke, of Berne, Deutsch Zeit. für Chir., Bd. 2, S. 380 | 7 | 5 |
| Stromeyer's Handbuch | 4 | 0 |
| Italian War, Schmidt's Jahrbücher, Bd. 156, S. 249 | 378 | 216 |
| Hosp. at Langensalza, Stromeyer | 7 | 1 |
| Statist. des Hôpit. de Paris, 1861—63 | 16 | 10 |
| Franco-German War, Schmidt's Jahrbücher, vol. 1856 | 31 | 12 |
| War of 1866, " " " | 7 | 1 |
| Totals | 2,477 | 683 |

Mortality, 28 per cent.

NOT STATED WHETHER, TRAUMATIC OR PATHOLOGICAL.

| AUTHORITIES. | CASES. | DEATHS. |
|---|---|---|
| Trans. Ill. State Med. Society, 1863 | 9 | 0 |
| Pennsylvania Hospital | 9 | 2 |
| K. k. allg. Krankenhaus, Wien | 72 | 23 |
| Leeds Infirmary, England, Mr. Nunnely | 62 | 22 |
| Edinburg Infirmary, 1857—68, { Dr. E. Gurlt, in Jahresbericht, Gesammt. Med., Bd. II. } | 21 | 12 |
| Glasgow " 1847—68, " " " | 101 | 38 |
| St. George's Hosp. Lon. 1864—68, " " " | 7 | 4 |
| Guy's Hosp., " 1861—68, " " " | 31 | 12 |
| London Hosp., " 1862—68, " " " | 31 | 15 |
| Middlesex " " 1867—68, " " " | 1 | 1 |
| Royal Free " " 1862—68, " " " | 6 | 2 |
| St. Mary's " " 1868, " " " | 1 | 0 |
| Arch. Klin. Chir. Bd. II, S. 261. Practice of Dr. Beck | 12 | 0 |
| " " " " XVII, S. 510—519 | 14 | 4 |
| " " " " X, S. 891 | 20 | 12 |
| Totals | 397 | 147 |

Mortality, 37 per cent.

FOR PATHOLOGICAL CAUSES.

| AUTHORITIES. | CASES. | DEATHS. |
|---|---|---|
| New York Hospital, Bost. Med. Jour., May 1, 1872 | 3 | 0 |
| Pennsylvania " " " " " " | 4 | 1 |
| Boston City " " " " " " | 5 | 1 |
| Mass. Gen. " " " " " " | 32 | 4 |
| Guy's " London, 1861—68 | 8 | 2 |
| St. George's " " 1864—68 | 6 | 1 |
| St. Thomas' " " | 2 | 0 |
| St. Bartholmew's " " 1853—71 | 42 | 6 |
| London " " 1862—68 | 5 | 1 |
| Middlesex " " 1867—68 | 1 | 1 |
| King's College " " 1863—68 | 4 | 2 |
| Royal Free " " 1862—68 | 1 | 0 |
| Westminster " " 1861—67 | 3 | 0 |
| St. Mary's " " 1868 | 1 | 0 |
| Edinburg Infirmary, 1859—68 | 19 | 7 |
| Glasgow " 1847—68 | 19 | 1 |
| Statist. des Hôpitaux de Paris, 1861—63 | 19 | 9 |
| Med. Reports, British Army | 3 | 0 |
| Archiv. Klin. Chirurg., Bd. VIII, S. 926, 928, 1088 | 4 | 2 |
| Deutsche Zeit. für Chir., Bd. II, S. 380 | 3 | 2 |
| Leeds Gen. Infirmary, Mr. Nunnely | 20 | 1 |
| Totals | 204 | 41 |

Mortality, 20 per cent.

GENERAL SUMMARY OF AMPUTATIONS OF THE ARM.

| | LAKE STATES. | | | ABROAD. | | |
|---|---|---|---|---|---|---|
| | CASES. | DEATHS. | PER CENT. MORT. | CASES. | DEATHS. | PER CENT. MORT. |
| Traumatic primary | 15 | 3 | 20 | 1,366 | 624 | 46 |
| " secondary | 8 | 0 | 0 | 507 | 232 | 46 |
| Pathological | 3 | 0 | 0 | 204 | 41 | 20 |
| Traumatic, time not stated, military cases | ...... | ...... | ...... | 2,477 | 683 | 28 |
| Time and cause not stated, nearly all civil practice | 1 | 0 | 0 | 397 | 147 | 37 |
| Totals, | 27 | 3 | 11 | 4,951 | 1,727 | 35 |

It appears, therefore, that amputations of the arm abroad have a mortality of 35 per cent., which is more than three times that of the Lake States. Civil cases abroad have also a much greater mortality than military ones, owing to the fact that soldiers are mostly young and vigorous men, and military amputations of the arm are largely done at once on the field of battle, before the patient has been subjected to the deadly miasm of the average military hospital.

(To be continued.)

